# The feasibility of delivering and evaluating stratified care integrated with telehealth (‘Rapid Stratified Telehealth’) for patients with low back pain: a feasibility and pilot randomised controlled trial

**DOI:** 10.1007/s10067-026-07955-w

**Published:** 2026-04-07

**Authors:** Andrew R. Gamble, Christopher Needs, Christopher G. Maher, Marnee J. McKay, David B. Anderson, Joshua M. Hutton, Tarcisio F. de Campos, Nadine E. Foster, David Martens, Danielle M. Coombs, Gustavo C. Machado, Christopher S. Han, Joshua R. Zadro

**Affiliations:** 1 Institute for Musculoskeletal Health, School of Public Health, Faculty of Medicine and Health, The University of Sydney, Sydney, NSW Australia; 2 Royal Prince Alfred Hospital, Sydney Local Health District, Sydney, NSW Australia; 3 Discipline of Physiotherapy, School of Health Sciences, Faculty of Medicine and Health, The University of Sydney, Sydney, NSW Australia; 4 Sydney Musculoskeletal Health, Faculty of Medicine and Health, The University of Sydney, Sydney, Australia; 5 Surgical, Treatment and Rehabilitation Service (STARS) Education and Research Alliance, The University of Queensland and Metro North Health, Brisbane, QLD Australia; 6 Centre for Innovation in Pain and Health Research (CIPHeR), School of Health and Rehabilitation Sciences, The University of Queensland, Queensland, Australia

**Keywords:** Feasibility, Low back pain, Pilot, Randomised controlled trial, Stratified care, Telehealth

## Abstract

**Objectives:**

Establish the feasibility of delivering and evaluating stratified care integrated with telehealth (‘Rapid Stratified Telehealth’) to reduce waiting times for people with low back pain seeking care at Australian public hospitals.

**Methods:**

We conducted a single-blinded, single site, 2:1 ratio, two-arm parallel feasibility and pilot randomised controlled trial (RCT) with nested qualitative interviews. Usual clinic-based care for low back pain was compared to Rapid Stratified Telehealth which matched the mode and type of care to participant’s risk of persistent disabling pain based on the Keele STarT MSK Tool and potential radiculopathy. Key process outcomes include acceptability of the model, intervention fidelity and adherence, appointment details, response, recruitment and consent rates, and missing data. Additional outcomes included waiting time to access care, clinical outcomes, healthcare utilisation, and adverse events. Quantitative outcomes were summarised descriptively. Qualitative data were analysed using thematic analysis.

**Results:**

Of 133 people screened, 101 were eligible (76%), and 40 (30%) were randomised (intervention 26, usual care 14). Feasibility targets were met for acceptability, fidelity, and missing data but not met for recruitment, consent, and response rates. Adherence data was uncertain due to poor reporting. Intervention participants waited a median of 13 days less for their first appointment vs. usual care participants (16 days vs. 29 days). Small sample size and differences in baseline characteristics mean additional outcomes should be interpreted with caution.

**Conclusion:**

This study provides important information to guide modifications to our Rapid Stratified Telehealth model of care and planning of a large multisite RCT across hospital outpatient clinics.

**Key Points**

**Table:** 

• *Our new model of care is feasible to deliver and evaluate in a fully powered RCT.* • *No intervention participant was at low risk of persistent disabling pain.* • *More than half of the intervention participants received clinic-based care.*

**Supplementary Information:**

The online version contains supplementary material available at 10.1007/s10067-026-07955-w.

## Introduction

Long waiting times in many Australian public hospitals prevent timely access to care for people with musculoskeletal conditions, including low back pain (LBP), which is the leading cause of disability in Australia and globally [1]. Over half of Australians (55%) do not have private health insurance, which can increase reliance on public care [2–5]. Delayed access to care for people with LBP can contribute to symptoms becoming more disabling, complex, and costly to manage [6–8]. A potential solution to reduce waiting times for people with LBP is a model of care that identifies LBP cases that can be managed with less resources (e.g. brief telephone appointments, self-management advice, App-based home exercise programmes), thereby freeing up clinic-based resources for more complex LBP cases.

‘PhysioDirect’ is a UK model of care that supported physiotherapists to triage patients with musculoskeletal conditions into needing simple telephone advice, education, and exercise instructions or clinic-based physiotherapist-led care [9]. PhysioDirect was compared to usual care where patients joined the usual waiting list for the next clinic-based appointment in a non-inferiority RCT across four physiotherapy services in England (*n* = 2249) [9]. PhysioDirect reduced waiting times (median of 7 days vs. 34 days in usual care) and the need for clinic-based appointments (40% relative reduction) compared to usual care, without compromising physical health outcomes. Qualitative interviews also found that PhysioDirect was acceptable to patients and physiotherapists in the trial [10]. This innovative overseas model of care inspired us to develop a similar one for the Australian context that could reduce long waiting times for musculoskeletal care.

In Australia, many public hospital outpatient clinics caring for patients with musculoskeletal conditions prioritise referrals based on the acuity of their presentation. For example, patients referred to outpatient physiotherapy clinics in New South Wales (NSW) are seen much sooner if they have acute musculoskeletal conditions (defined as symptoms less than 4 weeks) or require rehabilitation after surgery. Those with chronic musculoskeletal pain (defined as symptoms longer than 12 weeks) are usually not prioritised and often wait a long time to be seen in clinic. Unfortunately, acuity alone is not a good indicator of prognosis for many patients with musculoskeletal conditions (e.g. LBP) [11], and a shift to using validated prognostic tools could improve the way treatment is matched to patients [12].

In Australia, remotely delivered physiotherapy utilising telehealth has been shown to be non-inferior for improving patient function compared to clinic-based care in a recent randomised controlled trial for patients with musculoskeletal conditions (*n* = 210) [13]. Similarly, another randomised controlled trial conducted in Australia found that rehabilitation delivered via telehealth is non-inferior to clinic-based care for improving pain and function among people with chronic knee pain (*n* = 394) [13, 14]. Based on the PhysioDirect trial and recent telehealth and hybrid models of care in Australia, we proposed a new model of care for people with LBP referred to public hospital outpatient clinics. Our model integrates stratified care with telehealth (‘Rapid Stratified Telehealth’) and involves matching the mode and type of care patients are offered to their risk of persistent disabling LBP (based on the Keele STarT MSK Tool; low, medium, or high risk [15]) and presence of potential radiculopathy. The development of the model of care is detailed elsewhere [16]. In brief, we designed a preliminary model of care with a multidisciplinary steering group guided by research evidence [9, 10]. We then wanted to test the feasibility of the model of care with patients with LBP before considering broadening to other musculoskeletal conditions.

The aims of this feasibility and pilot RCT were to determine the (1) feasibility of delivering stratified care integrated with telehealth (‘Rapid Stratified Telehealth’) for patients with LBP referred to a public hospital outpatient clinic and (2) feasibility of a future multi-centre RCT to compare the clinical and cost-effectiveness of this new model compared to usual care for patients with various musculoskeletal conditions. Additional aims were to explore differences in waiting times, number of appointments, clinical outcomes (pain, function, quality of life, satisfaction), healthcare use, and adverse events between those who were managed with our ‘Rapid Stratified Telehealth’ model of care compared with usual care.

## Methods

### Study design

We conducted a single-blinded, single site, two-arm parallel feasibility and pilot RCT with nested qualitative interviews. Only outcome assessors collecting quantitative data were blinded when self-reported data were collected via telephone. The trial is reported in accordance with the CONSORT extension for randomised pilot and feasibility trials (Supplementary file 1) [17]. The nested qualitative interviews of clinician and patient acceptability of Rapid Stratified Telehealth are reported below according to the COREQ (Consolidated Criteria for Reporting Qualitative Research) (Supplementary file 2) [18].

### Participants and recruitment

The full methods are detailed in the feasibility and pilot RCT protocol [19]. In brief, participants were recruited from referrals to a multidisciplinary outpatient ‘Back Clinic’ at Royal Prince Alfred Hospital in Sydney, Australia, where rheumatologists assess patients and refer them to an outpatient physiotherapist if needed. The Back Clinic is usually open for two half days per week and staffed by four clinicians (one rheumatologist, one physiotherapist, and two registrars). For our trial, the head rheumatologist working at the Back Clinic screened new LBP referrals from primary care (e.g. general practitioners) according to our inclusion criteria (Box 1). Potentially eligible patients were then contacted by a physiotherapist researcher and informed they were on the waiting list for an appointment as per usual care. At the end of the routine call, the physiotherapist researcher mentioned the trial and screened the patient for eligibility, and if they agreed to participate, they were sent further information with the baseline questionnaires via email or post to provide consent (Supplementary file 3 and 4). All participants were informed that participation was voluntary, and non-consent to participate or withdrawing from the trial would not affect their care.

**Table:** Box 1 Inclusion and exclusion criteria

Inclusion criteria: ► 18 years or older ► LBP (non-specific LBP or radicular LBP/sciatica of any duration) ► New referral to the Back Clinic from primary care (i.e. have not been on the waiting list prior to enrolment) ► Willing to participate for up to 6 months and provide follow-up data at 6 weeks, 3 months and 6 months	
Exclusion criteria: ► Suspected serious underlying pathology (e.g., cancer, fracture, infection, inflammatory arthritis, cauda equina syndrome) ► Referral strongly suggestive of concerning neurological features (e.g., progressive radiculopathy)	

*LBP* low back pain

### Data collection

Baseline questionnaires collected data on demographic and clinical information prior to randomisation (Table 1). Participants completed baseline questionnaires via return paid envelopes or electronically in Research Electronic Data Capture (REDCap) via email, SMS, or telephone. Data entered into REDCap from hard copy questionnaires were double checked for accuracy, and assessors were blinded. The Keele STarT MSK tool [15] was completed by all participants as part of the baseline questionnaire to identify risk subgroups for the risk of developing persistent disabling pain. The STarT MSK tool consists of ten items covering domains about pain, self-management, function, well-being, and beliefs about activity and pain persistence [15]. The tool produces a total score out of 12, with subgroups scored as follows: 0–4 indicates low risk, 5–8 indicates medium risk, and 9–12 indicates high risk. We used the Keele STarT MSK tool instead of the Keele STarT Back tool [20] because we planned to include people with LBP and other musculoskeletal conditions in our future trial [21]. Due to clinician feedback, the protocol was modified before recruitment, so participants who reported pain that started from their back and went below their knee in the baseline questionnaire were classified as having ‘potential radiculopathy’ and offered clinic-based appointments. A single-item question at baseline was used to identify participants with potential radiculopathy instead of the clinician developed screening tool mentioned in our published protocol. This was because clinicians in the trial wanted a more sensitive measure to capture anyone with leg pain so they could perform a clinic-based neurological examination.

**Table 1:** Characteristics of participants

Demographics	Total sample (*n* = 40)	Intervention (*n* = 26)	Usual care (*n* = 14)
Age, median (IQR)^a^^	51 (39 to 66)^a^	55 (42 to 67)^c^	51 (29 to 59)^b^
Female, *n* (%)*	22 (56)	13 (52)	9 (64)
Language other than English spoken at home, *n* (%)*	6 (15)	5 (20)	1 (7)
Employment, *n* (%)*			
Currently employed	17 (44)	12 (48)	5 (36)
Not currently employed	17 (44)	11 (44)	6 (43)
Student	4 (10)	1 (4)	3 (21)
Unpaid carer	1 (3)	1 (4)	0 (0)
Education, *n* (%)*			
High school (not completed)	4 (10)	4 (16)	0 (0)
High school (completed)	6 (15)	4 (16)	2 (14)
TAFE/trade	12 (31)	5 (20)	7 (50)
University—undergraduate degree	8 (21)	5 (20)	3 (21)
University—postgraduate degree	7 (18)	6 (24)	1 (7)
Other	2 (5)	1 (4)	1 (7)
Symptom duration of 12 weeks or longer, *n* (%)*	39 (100)	25 (100)	14 (100)
Taken sick leave due to LBP, *n* (%)*	21 (54)	16 (64)	5 (36)
Keele STarT MSK Tool risk subgroup, *n* (%)			
Low risk (0 to 4)	1 (3)	0 (0)	1 (7)
Medium risk (5 to 8)	8 (20)	5 (19)	3 (21)
High risk (9 to 12)	7 (18)	5 (19)	2 (14)
Keele STarT MSK tool score (0 to 12), median (IQR)	9 (7 to 10)	9 (8 to 10)	9 (7 to 11)
Potential radiculopathy**	24 (60)	16 (62)	8 (57)

*N* number of participants who were randomised, *IQR* interquartile range, *TAFE* Technical and Further Education, *MSK* musculoskeletal. ^a^*n* = 37, ^b^*n* = 13, ^c^*n* = 24. ^Data on age were missing for two participants. *Data on gender, employment, education, and symptom duration and sick leave were missing for one participant from the intervention group. All percentage calculations for the total sample and intervention were based on a sample size of 39 and 25 respectively for gender, employment, education, and symptom duration and sick leave. **Pain that started from their back and went below their knee

### Interventions and procedures

#### Randomisation procedure

Consenting participants were randomised (2:1 ratio) to either the Rapid Stratified Telehealth model of care (intervention) or usual care. The 2:1 randomisation ratio was chosen to get more insight into the delivery of the intervention from a clinician, patient, and researcher perspective, improve the recruitment rate by making the trial more appealing to participants (i.e. a higher chance they would be allocated to the intervention), particularly since our recruitment site was only operating limited hours per week, and provide additional data on feasibility outcomes (e.g. recruitment, adherence) [17]. The random allocation sequence was independently generated in Stata statistical software and uploaded to REDCap. Allocation was concealed, and the physiotherapy researcher did not know the participant’s allocation until their baseline data were entered into REDCap. The allocation sequence was also concealed from participants and all staff associated with the trial. A secure computer-generated blocked random allocation sequence was generated using block sizes of 3, 6, or 9. Risk subgroup, as assessed by the Keele STarT MSK tool (low, medium, high risk), and the presence of potential radiculopathy (single item question in the baseline questionnaire) were used as stratification variables.

#### Rapid Stratified Telehealth and usual care

##### Rapid Stratified Telehealth (intervention)—subgroup allocation

Participants in the intervention group had their mode and type of care matched to their risk of persistent disabling pain, categorised as low, medium, or high (based on their baseline Keele STarT MSK score) or having potential radiculopathy (based on having pain that started from their back and went below their knee) [19]. The Keele STarT MSK tool was chosen due to its validity for identifying patients with musculoskeletal conditions at low, medium, or high risk of persistent disabling pain, which was demonstrated in a previous study conducted in the UK (*n* = 524) [15, 22]. This study included a large number of participants with low back pain (*n* = 155), highlighting the utility of the tool for participants recruited in this study [15, 22].

##### Rapid Stratified Telehealth (intervention)—matched treatment

All participants without potential radiculopathy in the intervention group received an initial call with the rheumatologist. Participants at low risk of persistent disabling pain were told they did not need further treatment as they were very likely to have a good prognosis and were given advice to gradually increase their daily walking (or other activities) as pain permitted, temporarily modify painful activities, and take a regular dose of paracetamol if required. They were also given written education material produced by the Agency for Clinical Innovation on managing low back pain (https://bit.ly/3iGfGrX). These participants were told to call back if their condition did not improve over the next 6 weeks. Participants at medium or high risk of persistent disabling pain were referred by the rheumatologist to physiotherapist-led telehealth (via videoconference). The number of telehealth sessions was determined by the treating physiotherapist (maximum of 12 sessions over 6 months; reflecting usual care in public hospitals). Physiotherapist-led telehealth included advice to support self-management and an exercise program prescribed using the app-based program PhysiTrack. Those in the high-risk group were additionally directed to complete an online self-directed pain education program developed by the Agency for Clinical Innovation (https://www.aci.health.nsw.gov.au/chronic-pain/for-everyone). Participants with potential radiculopathy had clinic-based sessions with a rheumatologist who took further medical history, conducted a physical and neurological examination, reviewed any previous investigations (e.g. imaging, pathology tests), formulated a management plan, and monitored progress. The rheumatologist determined the number of appointments they needed with a rheumatologist (maximum of 4 over 6 months; reflecting usual care). Participants with potential radiculopathy were also referred by the rheumatologist to clinic-based physiotherapist-led care (if needed) which included advice and education to support self-management, exercise therapy, graded activity, graded exposure, and/or spinal manipulative therapy. Treating physiotherapists ensured that participants with potential radiculopathy who were also at high risk of persistent disabling pain were offered interventions to address psychological barriers to recovery (e.g. psychologically informed physiotherapy) [23] and were referred to see a psychologist if necessary. The number of clinic-based physiotherapy appointments was determined by the treating physiotherapist (maximum of 12 over 6 months; reflecting usual care). Treatment recording forms and clinician notes were used to record the treatment provided.

##### Usual care

Participants in the usual care group were offered a clinic-based appointment with a rheumatologist. The rheumatologist took participants’ medical history, conducted a physical and neurological examination, reviewed any previously undertaken investigations (e.g. imaging, pathology tests), formulated a management plan, and monitored progress. The rheumatologist determined the number of appointments they needed (maximum of 4 over 6 months; reflecting usual care). If required, the rheumatologist referred participants to a physiotherapist, specialised pain clinic, or psychologist, consisted with usual care in the Back Clinic. If referred to a physiotherapist, management was determined by the treating physiotherapist and may have consisted of further advice and education to support self-management, exercise therapy, graded activity, graded exposure, and/or spinal manipulative therapy. The number of clinic-based appointments was determined by the treating physiotherapist (maximum of 12 over 6 months; reflecting usual care). Treatment recording forms and clinician notes were used to record the treatment provided.

### Outcomes

Feasibility measures (key process outcomes), feasibility targets, and additional outcomes are described in Box 2.

**Table:** Box 2 Feasibility measures (primary outcomes), feasibility targets, and secondary outcomes

Outcome measures	A priori feasibility targets
Key process outcomes:	
1. Feasibility outcomes for ‘delivering’ Rapid Stratified Telehealth:	
Clinician and patient acceptability of the intervention, determined using semi-structured interviews (see Sect. “Qualitative interview outcomes”)	Acceptable to clinicians and patients
Percentage of participants who are provided care that matches the protocol for their subgroup (‘intervention fidelity’), assessed via treatment recording forms	> 75%
Patient adherence to App-based exercises was only assessed for intervention group participants in the medium- and high-risk subgroups, as these were the only participants prescribed App-based exercises using PhysiTrack. Participants prescribed App-based exercises recorded adherence in the PhysiTrack App. Completion of the online pain education for participants in the high-risk subgroup was assessed via treatment recording forms	> 75%
Number of appointments, median appointment times, and attendance	No target
2. Feasibility outcomes for ‘evaluating’ Rapid Stratified Telehealth in a future multi-centred randomised controlled trial:	
Number of participants recruited per week over 6 months (recruitment rate)	≥ 3 participants per week
Number of eligible participants per week	No target
Percentage of participants who consent to be part of the study from those who were eligible (consent rate)	50% or more over 6 months, similar to the PhysioDirect trial [9]
Response rate at 6 weeks, 3 months, and 6 months	≥ 75%
Percentage of missing data for key outcome measures	< 15%
Additional outcomes:	
Treatment waiting time (i.e. median (IQR) duration in days from randomisation to first clinic-based or telehealth appointment with a rheumatologist). We note this may differ conceptually from the duration from referral to first clinic-based or telehealth appointment	
Clinical outcomes at 6 weeks, 3 months, and 6 months including Quality of life (PROMIS-29 Profile V.2.0 questionnaire; higher scores indicate better quality of life), pain (0–10 Numeric Rating Scale; 0 being no pain, 10 being the worst pain imaginable), disability (RMDQ 0–24 scale; higher scores indicate greater), and patient satisfaction (0–10 Numeric Rating Scale; 0 being the worst possible care, 10 the best care possible)	
Healthcare service and medication use at 6 weeks, 3 months, and 6 months	
Adverse events (AEs) at 6 weeks, 3 months, and 6 months: Adverse events were collected via self-reported surveys and categorised as a serious adverse event if they were life-threatening, resulted in hospitalisation, significant disability or incapacity, or death. Details of any reported adverse events were collected to determine if they were potentially related to the treatment provided	
Intervention and healthcare costs at 6 weeks, 3 months, and 6 months (to be reported in a separate paper)	

*PROMIS* Patient-Reported Outcomes Measurement Information System, *RMDQ* Roland and Morris Disability Questionnaire.

### Statistical analysis

The main analysis focused on the feasibility (process) of delivering Rapid Stratified Telehealth (acceptability, fidelity, adherence, and appointment details) and evaluating the model of care in a future multi-centre RCT (recruitment rates, consent rates, response rate, and percentage missing data). The a priori target sample size was 60 participants based on a rule of thumb for feasibility studies [19, 24]. Quantitative feasibility data were summarised using descriptive statistics (median and IQR, and counts and percentages, as appropriate). Qualitative feasibility data (acceptability) were analysed using Microsoft Excel (see the “Qualitative interviews” section).

The additional outcomes (see Box 2) were explored, comparing the Rapid Stratified Telehealth and usual care arms of the trial using descriptive statistics (median, IQRs, and counts and percentages, as appropriate). All quantitative analyses were conducted in STATA V.16.0. No statistical inference testing was performed as recommended by the CONSORT extension for randomised pilot and feasibility RCTs [17, 25]. The cost-effectiveness of the new model of care will be reported in a separate paper.

### Qualitative interviews

#### Participants and recruitment

Semi-structured interviews were conducted to investigate the acceptability of Rapid Stratified Telehealth. Interview participants were recruited via telephone or email using purposive sampling of patients and clinicians involved in the trial. Participants provided consent electronically in REDCap using a separate Participant Information Statement (Supplementary file 5) and Participant Consent Form (Supplementary file 6).

#### Data collection and analysis

One-on-one interviews were conducted via telephone or videoconference (e.g. Zoom) by one male physiotherapist researcher experienced in conducting semi-structured interviews and in qualitative research methods (AG). Additional verbal consent was gained from all participants before interviews were audio- or video-recorded. Semi-structured interview guides (Supplementary file 7) informed questioning on the pros and cons of stratification, telehealth, and barriers and facilitators to adapting our model of care for treating various musculoskeletal conditions in a larger multisite trial. Notes were taken during the interviews to highlight key themes that emerged and direct further questioning. Each interview was transcribed verbatim. Participants had the opportunity to review transcripts prior to data analysis.

All interview data were analysed using inductive thematic analysis, a method for identifying, analysing, and reporting patterns within data [26]. Two male physiotherapist researchers (AG and CH) independently familiarised themselves with interviews (via audio-recordings and transcripts), recorded initial observations, and identified concepts relevant to the questions asked. The two researchers (AG and CH) developed a framework to organise concepts into broader themes and sub-themes in Microsoft Excel. Any disagreements in categorising concepts into themes and sub-themes were discussed and resolved with a third researcher (JZ). The mapping of themes and sub-themes was an iterative process as the new data emerged. Interviews were stopped once no new themes were identified in three consecutive interviews (data saturation). Interview themes were matched to two key outcomes: (1) the feasibility of delivering Rapid Stratified Telehealth, (2) the potential to adapt the model for other musculoskeletal conditions. Interview data from this study has been combined with further interview data and analysed further in a separate paper [16].

## Results

### Participant flow and recruitment

**Fig. 1:**
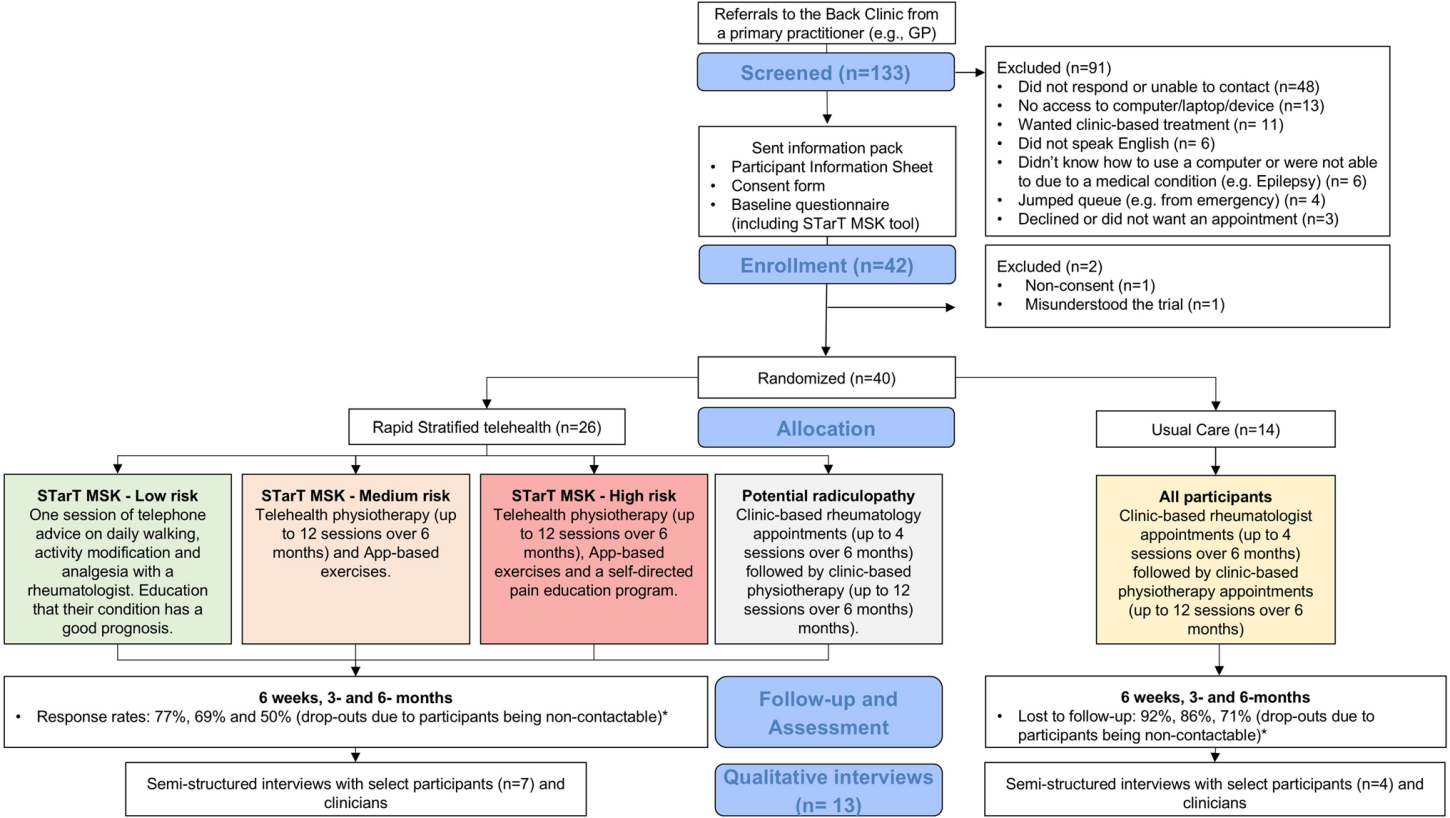
Consort flow diagram

Of the 133 patients screened between July 2021 and March 2024, 101 were eligible to participate, and 40 consented and were randomised (40% consent rate; Fig. 1, Table 2). The overall recruitment rate was 0.4 participants per week (recruited over 95 weeks) and, if converted to reflect a clinic that was open 5 days a week from 9 to 5 pm, the recruitment rate would have been 4 participants per week (Table 2). All participants were referred to the hospital clinic by their general practitioner. Recruitment was stopped before reaching the planned sample size (*n* = 60) as the authorship team believed sufficient data had been obtained to investigate the primary feasibility objectives of this study [17]. Recruitment was also slower than expected due to a reduction in staff and opening hours at the Back Clinic following the COVID-19 pandemic, so we decided to stop recruitment at 40 participants as we believed the added cost of continuing the trial to recruit an extra 20 participants would not correspond to more insightful feasibility data.

**Table 2:** Key feasibility outcomes

Outcome measures	Feasibility target met: yes/no
1. Feasibility outcomes for ‘delivering’ Rapid Stratified Telehealth (intervention):	
Qualitative interviews: Patients and clinicians suggested the model of care and trial were acceptable (see the “Qualitative interview outcomes” section)	Yes
Intervention fidelity: *Percentage who received matched physiotherapist-led care in the intervention group:* • 26 total participants in the intervention group • 16/26 were referred to physiotherapy; 6/16 did not attend any appointment • Of the 10 that attended physiotherapy appointments over the study period, 8 (80%) received matched physiotherapist-led care: • Medium risk; 1/2 (50%) • High risk; 1/1 (100%) • Potential radiculopathy; 6/7 (86%)	Yes
Intervention adherence: • App-based exercises: 2/10 participants in the medium- and high-risk groups were prescribed app-based exercises, and no participant reported adherence via PhysiTrack • Online pain education program: 1/5 participants in the high-risk group reported completing the program as per the treatment recording forms, 4/5 participants did not have treatment recording forms as 2/5 participants were not given a physiotherapy appointment and 2/5 did not attend their physiotherapy appointment	Uncertain
Number of appointments: *Median (IQR) number of rheumatology appointments*: • Intervention 1 (1 to 2) vs. usual care 1 (1 to 2) *Total number of rheumatology appointments:* • Intervention 21 vs. usual care 16 *Median (IQR) number of physiotherapy appointments:* • Intervention 3 (1 to 8) vs. usual care 7 (5 to 8) *Total number of physiotherapy appointments:* • Intervention 44 vs. usual care 45 Appointment times for all participants: *Median (IQR) appointment times*: • Rheumatology appointments 60 min (60 to 60), intervention 60 min (45 to 60), vs. usual care 60 min (60 to 60) • Physiotherapy appointments 60 min (60 to 60), intervention 60 min (60 to 60) vs. usual care 60 min (60 to 60) Attendance for all participants: *Number of participants referred for physiotherapy*: • 26/40 (65%) were referred for physiotherapy, 16/26 (62%) intervention participants and 10/14 (71%) usual care participants • 17/26 (65%) attended at least one appointment overall, 10/16 (63%) intervention participants vs. 7/10 (70%) usual care participants	-
2. Feasibility outcomes for ‘evaluating’ Rapid Stratified Telehealth (intervention) in a future multi-centred RCT:	
Recruitment rate: *Participants per week:* • 0.4 recruited over 95 weeks	No*
Consent rate: *Percentage of eligible participants:* • 40% (40 participants from 101 eligible)	No
Response rate: *Percentage completing follow up surveys:* • 82% at 6 weeks, 75% at 3 months, and 57% at 6 months	No
Missing data: *Percentage at baseline:* • 2/40 participants had missing data on age (5%), 1/40 participant for gender (3%), employment (3%), education (3%), symptom duration (3%) and sick leave (3%)	Yes

*The criterion was met if the recruitment rate was converted to reflect a clinic that was open 5 days a week from 9 to 5 pm during the study period

### Characteristics of trial participants

Participants’ median age (interquartile range, IQR) was 51 (39 to 66) years and 56% were female. Participants in the Rapid Stratified Telehealth group were slightly older (median age 55 vs. 51 years old), more frequently reported speaking a language other than English at home (20% vs. 7%), and almost twice as likely to have taken sick leave due to their LBP (64% vs. 36%). All participants had symptoms for 12 weeks or longer.

#### Primary outcomes and feasibility targets

Three out of seven pre-defined feasibility targets were met (Table 2), and one we deemed as uncertain (adherence data).

##### Additional outcomes

*Treatment waiting time*

Median (IQR) waiting time of duration in days from randomisation to first clinic-based or telehealth appointment with a rheumatologist was 16 days (7 to 30) in the intervention group and 29 days (7 to 89) in the usual care group.

*Health service and medication use*

Health service use outside the trial was similar between the intervention and usual care groups at 6 weeks and 3 months and higher in the intervention group at 6 months. Over half of the trial participants reported seeking healthcare that was ongoing. The number of participants using medications for their LBP was similar between the intervention and usual care groups at 6 weeks, lower in the intervention group at 3 months, and similar at 6 months (Table 3).

*Clinical outcomes, patient satisfaction, and adverse events*

Median pain intensity and satisfaction ratings were similar between participants in the intervention and usual care groups at 6 weeks, 3 months, and 6 months. Median quality of life using the 29-item Patient-Reported Outcomes Measurement Information System (PROMIS-29) was lower in the intervention group compared to the usual care group at 6 weeks and 3 months and similar between groups at 6 months; higher scores indicate better quality of life. Median disability using the Roland and Morris Disability Questionnaire (RMDQ) was higher in the intervention compared to usual care groups at 6 weeks, similar at 3 months, and higher in the intervention group at 6 months; higher scores indicate greater disability. Adverse event rates were similar between the intervention and usual care groups at 6 weeks, higher in the intervention group at 3 months, and similar at 6 months. No serious adverse event occurred during the study (a life-threatening event or event resulting in hospitalisation, significant disability or incapacity, or death) (Table 3).

**Table 3:** Additional feasibility RCT outcomes

Outcome	Total		Intervention		Usual care	
	Median (IQR)	No. of participants	Median (IQR)	No. of participants	Median (IQR)	No. of participants
PROMIS-29						
Physical function (0–20)						
Baseline	13 (11 to 17)	40	12 (8 to 15)	26	13 (11 to 17)	14
6 weeks	14 (10–16)	33	12 (10 to 16)	20	15 (11 to 18)	13
3 months	13(10 to 16)	30	12 (10 to 15)	18	16 (12 to 18)	12
6 months	13 (11 to 16)	23	13 (9 to 14)	13	15 (11 to 17)	10
Anxiety (0–20)						
Baseline	12 (7 to 14)	40	11 (7 to 13)	26	11 (4 to 15)	14
6 weeks	11 (6 to 12)	33	11 (8 to 13)	20	11 (5 to 12)	13
3 months	10 (5 to 12)	30	11 (5 to 14)	18	10 (5 to 11)	12
6 months	9 (5 to 12)	23	9 (4 to 12)	13	9 (6 to 12)	10
Depression (0–20)						
Baseline	10 (5 to 13)	40	9 (5 to 13)	26	11 (5 to 13)	14
6 weeks	10 (6 to 12)	33	10 (6 to 12)	20	11 (6 to 12)	13
3 months	10 (4 to 12)	30	10 (4 to 12)	18	10 (5 to 12)	12
6 months	8 (4 to 12)	23	8 (4 to 12)	13	7 (4 to 11)	10
Fatigue (0–20)						
Baseline	12 (10 to 17)	40	13 (10 to 17)	26	11 (9 to 17)	14
6 weeks	12 (9 to 16)	33	12 (9 to 17)	20	11 (8 to 16)	13
3 months	13 (9 to 16)	30	13 (12 to 17)	18	10 (9 to 12)	12
6 months	11 (7 to 15)	23	14 (9 to 16)	13	9 (7 to 11)	10
Sleep disturbance (0–20)						
Baseline	13 (10 to 16)	40	13 (10 to 16)	26	12 (10 to 15)	14
6 weeks	11 (9 to 16)	33	12 (10 to 16)	20	11 (9 to 16)	13
3 months	12 (9 to 16)	30	12 (10 to 16)	18	13 (9 to 16)	12
6 months	11 (8 to 14)	23	11 (9 to 14)	13	11 (7 to 14)	10
Social participation (0–20)						
Baseline	9 (3 to 12)	40	7 (3 to 13)	26	10 (3 to 12)	14
6 weeks	9 (4 to 14)	33	9 (1 to 15)	20	12 (4 to 13)	13
3 months	12 (3 to 15)	30	11 (3 to 14)	18	12 (5 to 16)	12
6 months	12 (3 to 15)	23	10 (3 to 12)	13	13 (7 to 15)	10
Pain interference (0–20)						
Baseline	14 (12 to 16)	40	14 (12 to 16)	26	13 (12 to 16)	14
6 weeks	14 (9 to 16)	33	16 (9 to 16)	20	9 (8 to 16)	13
3 months	13 (8 to 15)	30	14 (8 to 15)	18	11 (7 to 14)	12
6 months	14 (8 to 15)	23	15 (12 to 15)	13	10 (8 to 14)	10
RMDQ total (0–24)						
Baseline	12 (7 to 14)	40	12 (9 to 14)	26	12 (4 to 14)	14
6 weeks	10 (5 to 14)	33	12 (5 to 15)	20	8 (5 to 14)	13
3 months	9 (6 to 13)	29	10 (6 to 11)	17	9 (6 to 16)	12
6 months	12 (3 to 16)	23	12 (8 to 14)	13	9 (3 to 16)	10
Pain in the past 7 days (0–10)						
Baseline	6 (5 to 7)	40	6 (5 to 7)	26	6 (4 to 6)	14
6 weeks	5 (4 to 6)	33	6 (5 to 6)	20	5 (3 to 6)	13
3 months	5 (4 to 6)	29	5 (4 to 6)	17	5 (3 to 5)	12
6 months	6 (5 to 7)	22	6 (6 to 7)	12	6 (4 to 7)	10
Satisfaction with care (0–10)						
Baseline	Not assessed	Not assessed	Not assessed	Not assessed	Not assessed	Not assessed
6 weeks	5 (2 to 7)	33	5 (2 to 8)	20	5 (1 to 7)	13
3 months	5 (2 to 8)	29	5 (1 to 6)	17	6 (4 to 9)	12
6 months	6 (3 to 8)	22	5 (0 to 6)	12	8 (6 to 8)	10
Health service use	Yes (%)	No. of participants	Yes (%)	No. of participants	Yes (%)	No. of participants
Baseline	36 (90)	40	23 (89)	26	13 (93)	14
6 weeks	27 (82)	33	16 (80)	20	11 (85)	13
3 months	24 (83)	29	14 (82)	17	10 (83)	12
6 months	19 (86)	22	11 (92)	12	8 (80)	10
Ongoing health service use	Yes (%)	No. of participants	Yes (%)	No. of participants	Yes (%)	No. of participants
Baseline	19 (48)	40	15 (58)	26	4 (29)	14
6 weeks	19 (58)	33	13 (65)	20	6 (46)	13
3 months	18 (62)	29	10 (59)	17	8 (67)	12
6 months	16 (73)	22	8 (67)	12	8 (80)	10
Medication use	Yes (%)	No. of participants	Yes (%)	No. of participants	Yes (%)	No. of participants
Baseline	31 (78)	40	21 (81)	26	10 (71)	14
6 weeks	23 (70)	33	15 (75)	20	8 (62)	13
3 months	19 (66)	29	12 (71)	17	7 (58)	12
6 months	17 (77)	22	9 (75)	12	8 (80)	10
Adverse events*	Yes (%)	No. of participants	Yes (%)	No. of participants	Yes (%)	No. of participants
Baseline	Not assessed	Not assessed	Not assessed	Not assessed	Not assessed	Not assessed
6 weeks	8 (24)	33	5 (25)	20	3 (23)	13
3 months	6 (21)	29	5 (29)	17	1 (8)	12
6 months	12 (55)	22	6 (50)	12	6 (60)	10

*IQR*, interquartile range; *No.*, number; Quality of life (PROMIS-29 Profile V.2.0 questionnaire; Patient-Reported Outcomes Measurement Information System: 0–10 pain score and a 5-point Likert Scale for seven other health domains where higher scores indicate better quality of life), pain (measured using a 0–10 Numeric Rating Scale; 0 being no pain to 10 being the worst pain imaginable); *RMDQ*, Roland and Morris Disability Questionnaire (scale 0–24; high scores indicate greater disability), and patient satisfaction (11-point numerical scale; 0 being the worst possible care and 10 the best care possible). *Adverse events included musculoskeletal pain (e.g. knee or Achilles injury), hernia, fractures (e.g. ankle), worsened pain intensity, location or frequency of pain (e.g. shoulder, upper back or radiating pain), nausea, dizziness, COVID-19, infection, vascular problems in the lower limb, or planned surgery.

### Qualitative interview outcomes

We conducted 13 interviews with 11 patients (*n* = 7 in the intervention group, *n* = 4 usual care) and 2 clinicians involved in the trial (*n* = 1 rheumatologist, *n* = 1 physiotherapist), lasting between 30 and 50 min. Characteristics of interviewed participants are outlined in Supplementary file 8. Qualitative interviews were summarised in seven key themes and described in more detail in another paper [16].

#### Feasibility of delivering the Rapid Stratified Telehealth model

Management of patients using the new model of care was seen as a safe solution to reducing waiting times in a currently overloaded system by clinicians. Clinicians suggested that all patients would benefit from an initial telephone assessment to build relationships between the clinician and patient and empower self-management aligned with evidence-based care. Clinicians suggested it can be time-consuming to review a referral, call and assess a patient, and make a booking, but if there are sufficient staff and support, there is potential to see patients sooner and provide clinic-based care to those who need it most. Some patients were apprehensive about not having physical touch via telehealth or trusting the accuracy of a remote assessment compared to clinic-based appointments, but all participants acknowledged the convenience of the intervention. Those who were managed via telehealth suggested their overall experience was acceptable and using the App-based exercise program PhysiTrack improved their ability to learn exercises and track progress. The ease of access and knowledge to use necessary equipment and technology was suggested to influence acceptability of the new model of care.

*They appreciated being contacted as they can often wait for a year and so they felt like they hadn't been forgotten. (Clinician, M, 60–70 years old)*

*It was quite strange initially, but then I got used to it and looked forward to it.*

*(Patient, F, unknown years old)*

*The convenience outweighs the need for personal touch. (Patient, M, 50–60 years old)*

*Sometimes the technological issues could be one of the barriers.*

*(Clinician, F, 20–30 years old)*

#### Potential to adapt the Rapid Stratified Telehealth model for other musculoskeletal conditions

The intervention was frequently suggested to be acceptable by patients and clinicians involved in the trial to manage other musculoskeletal conditions. Beliefs about anatomical complexity, pain characteristics, and the ability to adapt specific exercises for various conditions influenced perceived adaptability. All participants agreed that patients whose mobility was impacted would be appropriate for remote care irrespective of their diagnosis. Clinicians suggested that patients with any musculoskeletal conditions managed with evidence-based advice and exercise would be suited to remote care.

*If I was at home and house bound, I definitely think I would benefit from that opportunity (Patient, M, 50–60 years old)*

*There is potential for osteoarthritis to be managed using virtual care. (Clinician, M, 60–70 years old)*

*The ankle seems more complex and could be difficult to assess online. (Patient, F, unknown years old)*

## Discussion

### Summary of findings

Less than half of the feasibility targets we set a priori were met. Qualitative interviews suggested the Rapid Stratified Telehealth model of care was acceptable and offered convenience to access advice and treatment sooner. The a priori targets not met included recruitment and consent rates, and response rates, which should be interpreted with caution. Recruitment and consent rates were not met largely due to a reduction in staff and operating days of the Back Clinic following the COVID-19 pandemic. The low response rate at 3- and 6-months limits our ability to determine the longer-term impact of the intervention group compared to usual care. However, it provided valuable learnings for implementing retention strategies to improve follow-up response rates in a future definitive trial (e.g. participant reimbursement for completing follow-up surveys, regular trial updates to keep participants engaged). Adherence to the model of care was uncertain due to low appointment attendance, which also occurred for participants receiving usual care. Qualitative interviews suggested that relatively modest attendance to clinic-based physiotherapy appointments could be partly due to parking fees in the local areas and transport costs. Participants in the intervention group waited 13 days less for their first appointment with the rheumatologist compared to the usual care group, despite no participants in the intervention group receiving the low-risk treatment pathway (i.e. a single telephone appointment). Participants in both groups had poor attendance to allocated physiotherapy appointments and largely similar clinical outcomes (i.e. pain, satisfaction). Participants in the intervention group had fewer appointments compared to the usual care group but reported slightly higher disability ratings at all follow-ups.

### Comparison to existing research

Our findings align with previous qualitative studies indicating the acceptability of remotely delivered care (e.g. telehealth) [27, 28] and high-quality RCTs in Australia showing that physiotherapist-led telehealth is as effective as usual clinic-based care for people with a variety of musculoskeletal conditions [13] including osteoarthritis [29]. Participants we interviewed provided positive feedback about the convenience of attending telehealth appointments and using App-based exercises within our model. Clinicians suggested a telephone call or telehealth appointment was acceptable to provide advice and support for self-management and could be used for patients with a variety of musculoskeletal conditions. A recent systematic review of patients with various musculoskeletal conditions (*n* = 845) also strengthens the argument that mobile Apps can improve clinical outcomes compared to exercises interventions without app support [30]. High non-attendance to physiotherapy appointments found in our trial aligns with a recent narrative review suggesting this is a global problem occurring due to various factors including age, gender, marital status, education, distance to hospital, and referral source [31]. For example, people are less likely to attend appointments if they are younger, male, not married, or referred by a general practitioner rather than a specialist [31]. The study also found higher education levels are associated with lower non-attendance rates [31].

### Implications for future evaluation

Our findings suggest that modifications to the model of care and analysis of the single site processes could enhance future evaluation. A multidisciplinary Back Clinic was selected as the single site for this trial due to the clinics limited capacity for new appointments, long waiting times for referred patients, and enthusiasm from the rheumatologists and physiotherapists at this site to test a model of care that addressed these issues. We found wait times to first appointment with a rheumatologist were reduced for participants in the Rapid Stratified group compared to usual care. Our model was designed to use a single telephone call for patients at low risk of persistent disabling pain to provide reassurance and support for self-management, without further treatment. Discharging low-risk patients remotely after one session would allow the next patient on the waiting list to be contacted sooner. No participants in the intervention group were classified as being at low risk of persistent disabling pain, meaning we were unable to realise the benefit (from an efficiency and waiting time perspective) of a single telephone call to provide advice and support to these participants. Ensuring recruitment of participants at low risk of developing persistent disabling symptoms and use of a more valid screening algorithm for radiculopathy in future trials could help better determine between-group differences in waiting time and clinical outcomes. Nevertheless, we suspect the reduction in waiting time we found was due to medium- and high-risk participants who were encouraged to self-manage via an exercise App and use less staffing resources.

To address recruitment, consent, and response rates, our future adequately powered trial evaluating this model of care is being conducted at multiple sites with more health professionals and longer operating hours, and we have funding to more regularly follow up participants and offer compensation for completing follow-up surveys. We are also involving sites that are more likely to include participants who would be stratified into all risk subgroups, which may reduce the number of participants classified as having ‘potential radiculopathy’ and lead to a larger reduction in waiting times by more participants utilising the low-risk management pathway. We are also including sites that include participants who have symptoms of various durations and using the duration of symptoms as a stratification variable. Future research could also investigate how the STarT MSK tool can be used during primary care before referral to hospital outpatient musculoskeletal clinics to triage patients sooner [32]. Future implementation success of our model will likely depend on factors such as individual patient characteristics, the complexity of different musculoskeletal conditions, and the acceptability of telehealth by clinicians and patients.

### Strengths and limitations

Our study had several strengths. We conducted a feasibility and pilot RCT with concealed allocation and reported it according to CONSORT extension for randomised pilot and feasibility RCTs [17]. The nested qualitative interviews of patient and clinician acceptability of our new model of care were reported according to the COREQ checklist (Consolidated Criteria for Reporting Qualitative Research) [18]. The model of care protocol was developed prior to recruitment with patients with LBP and clinicians involved in managing these patients and is described in more detail elsewhere [16].

The study has several important limitations. It was conducted at a single site with a complex patient population, which provided challenges in terms of recruitment, risk stratification, and response rates, but also provided valuable learnings to modify the protocol for evaluation in our future large multisite trial [21]. For example, during the COVID-19 pandemic, the Back Clinic was reduced to one rheumatologist and one physiotherapist, and only open for half a day, once per week, which significantly impacted the speed of recruitment due to limited time available to review new referrals. However, interviews with these clinicians suggested that settings with administration support could help to reduce the time needed to book appointments and lead to substantially faster recruitment. The low response rate at 3- and 6-month follow-ups also limited our ability to determine the longer-term impact of the intervention group compared to usual care. Given the small sample size and differences in baseline characteristics (e.g. taken sick leave due to LBP; intervention (64%) vs. usual care (36%)), the interpretation of patient outcomes requires caution. The use of a single question to identify participants with ‘potential radiculopathy’ likely has limited diagnostic accuracy and increased the proportion of participants allocated clinic-based appointments. However, clinicians involved in the trial liked this measure as it was more sensitive (as compared to our a priori detailed assessment) to capture anyone with leg pain so they could perform a neurological examination to identify radiculopathy in person. Recruitment of participants from a multidisciplinary outpatient ‘Back Clinic’ instead of an outpatient physiotherapy clinic may have limited the number of patients classified at low risk of persistent disabling pain, as all participants were referred to the clinic because it was deemed necessary that they see a rheumatologist. The use of the Keele STarT MSK tool instead of the STarT Back tool may have impacted the number of participants classified as low risk, as it has several domains not included in the STarT Back tool [33]. Nevertheless, the validation study for the STarT MSK included 155 participants with low back pain, suggesting it is still fit for purpose for participants in this trial [15]. We found similar pain and satisfaction ratings between participants in the intervention and usual care groups, but higher disability ratings for participants in the intervention group. Our large, adequately powered future RCT will use a non-inferiority design to better establish the effect of our new model on clinical outcomes.

## Conclusion

This study found that our Rapid Stratified Telehealth model of care is largely feasible to deliver and evaluate in a future fully-powered RCT. Key process outcomes provided important information to guide modifications to our model of care and trial processes for a large multisite RCT in outpatient hospital settings that manage patients with a variety of musculoskeletal conditions. Participants in the intervention group waited 13 days fewer for their first appointment with a rheumatologist compared to the usual care group, but the additional outcomes should be interpreted with caution due to a small sample size and differences in baseline characteristics.

## Supplementary Information

Below is the link to the electronic supplementary material.

ESM 1 (DOCX 162 KB)

ESM 2 (PDF 480 KB)

ESM 3 (PDF 40.4 KB)

ESM 4 (PDF 197 KB)

ESM 5 (DOCX 68.7 KB)

ESM 6 (DOCX 62.1 KB)

ESM 7 (DOCX 38.0 KB)

ESM 8 (DOCX 17.4 KB)

## Data Availability

The authors have full control of all primary data and agree to allow the journal to review their data if requested.
