# Supplementary material for: The feasibility of delivering and evaluating stratified care integrated with telehealth (‘Rapid Stratified Telehealth’) for patients with low back pain: a feasibility and pilot randomised controlled trial
Source: Clin Rheumatol. 2026 Apr 7;45(6):3771–84. doi: 10.1007/s10067-026-07955-w (PMC13249632; doi:10.1007/s10067-026-07955-w)
Supplement: Supplementary file 3 — (PDF 40.4 KB) [file 10067_2026_7955_MOESM3_ESM.pdf]

Participant Consent Form

Please complete the survey below.

Thank you!

Rapid Virtual Stratified Care: a feasibility trial comparing two care pathways for people referred to the Back Clinic: Participant Consent Form for Interviews

---

I (Your name), ______
Of (Your full address), ______
Email address

______
and have discussed the study with (name of investigator responsible for conducting informed consent)

______

Please download a copy of the "Participant Information Sheet" for your records.

[Attachment: "PARTICIPANT INFO SHEET.pdf"]

I have read and understood the 'Participant Information Sheet' on the above named research study — ◯ Yes ◯ No

---

If no, please contact the research team to discuss any concerns before proceeding to the survey on agam1165@uni.sydney.edu.au or (02) 8627 7423

I would like to receive a copy of the study results when they become available
◯ Yes
◯ No

I have been made aware of the procedures involved in the study, including any known or expected inconvenience, risk, discomfort or potential side effect and of their implications as far as they are currently known by the researchers. I understand that my de-identified data may be used for future research and I agree to this. I understand that, during the course of this study, my medical records may be accessed by Sydney Local Health District by regulatory authorities or by the Ethics Committee approving the research in order to verify results and determine that the study is being carried out correctly. I understand that the SLHD software license for REDCap (Research Electronic Data Capture) will be used to manage the collection and storage of my research data. I have had an opportunity to ask questions and I am satisfied with the answers I have received. I freely choose to participate in this study and understand that I can withdraw at any time. I consent to the future use of any data / samples I provide for research purposes. I understand that before they can use any data I provide, they must seek additional ethics approval. I consent for other research collaborators to use any data / samples I provide for future research purposes. I understand that before they can use my data, they must seek additional ethics approval. I also understand that the research study is strictly confidential. I consent to the storage and use of my information collected from me for use, as described in the relevant section of the Participant Information Sheet, for: This specific research project Other research that is closely related to this research project Any future research I hereby agree to participate in this research study.

◯ Yes I would be happy to continue and complete the survey
◯ No I would prefer not to complete the survey

Participant signature

____

Date of consent

____
