# Supplementary material for: The feasibility of delivering and evaluating stratified care integrated with telehealth (‘Rapid Stratified Telehealth’) for patients with low back pain: a feasibility and pilot randomised controlled trial
Source: Clin Rheumatol. 2026 Apr 7;45(6):3771–84. doi: 10.1007/s10067-026-07955-w (PMC13249632; doi:10.1007/s10067-026-07955-w)
Supplement: Supplementary file 4 — (PDF 197 KB) [file 10067_2026_7955_MOESM4_ESM.pdf]

THE UNIVERSITY OF SYDNEY

School of Public Health
Faculty of Medicine and Health

ABN 15 211 513 464

Dr Joshua Zadro
Chief Investigator
Research Fellow

Room 10/071
Level 10 North, King George V Building
Royal Prince Alfred Hospital
The University of Sydney
NSW 2050 AUSTRALIA
Telephone: +61 2 8627 6782
Facsimile: +61 2 8627 6262
Email: joshua.zadro@sydney.edu.au
Web: http://www.sydney.edu.au

Rapid Virtual Stratified Care:
a feasibility trial comparing two care pathways for people referred to the Back Clinic

PARTICIPANT INFORMATION STATEMENT

1. What is this study about?

You are invited to take part in a research study that will explore a new care pathway for people with back pain and/or leg pain radiating from the back. This Participant Information Statement tells you about the study. Knowing what is involved will help you decide if you want to take part. Please read this sheet carefully and ask questions about anything that you don't understand or want to know more about.

Participation in this research study is voluntary.

By giving your consent to take part in this study you are telling us that you:

- ✓ Understand what you have read
- ✓ Agree to take part in the research study as outlined below
- ✓ Agree to the use of your personal information as described

This Participant Information Statement is yours to keep.

Currently, when you are referred to see a Rheumatologist or Physiotherapist at Royal Prince Alfred Hospital's 'Back Pain Clinic', you are placed on a waiting list. Unfortunately, waiting times for treatment are currently 3 months or longer. This is referred to 'usual care', which is the care you would normally receive when referred to the 'Back Pain Clinic'. Our project involves testing a new pathway using telephone and virtual appointments, and an App-based exercise program. This new pathway is based on 'stratified care'. This involves matching the type and amount of care you receive based on your risk of persisting pain and presence of other symptoms (like leg pain). We want to see whether the new pathway helps people receive treatment sooner and recover sooner.

To find out which pathway is best, we will offer one third of people the current pathway and two-thirds the new pathway. We will monitor the two groups for 6 months and compare what happens between the groups. To ensure the groups are as similar to each other as possible, the group that you will be placed into is by chance. **There is a 67% chance you will be managed according to the new pathway, and a 33% chance you will be managed according to the current pathway.** To make the results of our study fair, we will not tell you which pathway you have been allocated to.

If you decide you would not like to participate in the research study, you will be managed according to the current pathway. However, your decision whether to participate will not affect your current or future relationship with the researchers or anyone else at the University of Sydney or Royal Prince Alfred Hospital. It also won't affect your position on the waiting list or the quality of care you receive.

2. Who is running the study?

This study is funded by the Agency for Clinical Innovation (ACI) New South Wales and the National Health Medical Research Council. Neither funder will benefit commercially from this study. The manufacturers of PhysiTrack, the mobile App you may be provided during the study, do not have any commercial, financial or business interests in this study.

The people conducting this study are:

- Dr Joshua Zadro, NHMRC Postdoctoral Researcher, Institute for Musculoskeletal Health University of Sydney and Sydney Local Health District
- Dr Chris Needs, Staff Specialist Rheumatologist, Royal Prince Alfred Hospital, Sydney Local District Health
- Prof Christopher Maher, Director, Institute for Musculoskeletal Health, University of Sydney and Sydney Local Health District
- Dr David Martens, Rheumatologist Advanced Trainee, Royal Prince Alfred Hospital, Sydney Local District Health
- Ms Danielle Coombs, Physiotherapist, Institute for Musculoskeletal Health University of Sydney and Sydney Local Health District
- Dr Gustavo Machado, NHMRC Postdoctoral Researcher, Institute for Musculoskeletal Health University of Sydney and Sydney Local Health District
- Mrs Charlotte McLennan, Network Manager, Institute for Musculoskeletal Health University of Sydney and Sydney Local Health District
- Dr Cameron Adams, Rheumatologist Advanced Trainee, Royal Prince Alfred Hospital, Sydney Local District Health
- Prof Nadine Foster, Director, Surgical, Treatment and Rehabilitation Service (STARS) Research and Education Alliance, The University of Queensland and Metro North Hospital and Health Service

3. Who can take part in the study?

A person will be allowed to participate in this study if he or she:

- is referred to the 'Back Pain Clinic' at Royal Prince Alfred Hospital
- has low back pain and/or leg pain radiating from the back
- is 18 years or over and able to provide informed consent

4. What does the study involve?

If you agree to participate in our study, we will send you a survey asking questions about you and your low back pain. We kindly ask you to complete these questionnaires and return them back to us via mail (return-paid envelope provided), email, or SMS. After this, you will be randomly allocated (i.e. by chance) to be managed using the new pathway or current pathway. We will send you another questionnaire at 6 weeks, 3 months, and 6 months after joining the study to see how your low back pain has changed. This questionnaire will contain similar questions to the first one you will complete. If you desire any more information at any point of the study, relevant contact details will be provided.

After 6 months, we may contact you to participate in a group interview (with up to 8 other participants) or one-on-one interview if you prefer. This interview may be conducted via telephone or videoconference

(e.g. Zoom) or in person at the Institute for Musculoskeletal Health, Level 10 King George V Building, Royal Prince Alfred Hospital. The interview will explore your opinions on the care you received. You will be sent more information about this interview before you agree to participate.

5. How much of my time will the study take?

If you decide to participate, your treatment time is unlikely to be different than if you did not participate and joined the current waiting list. However, by participating in the study, we will ask you to complete one survey when you enter the study, and another at 6 weeks, 3 months, and 6 months. Each survey will take between 10-15 minutes. You may also be asked to participate in a 1-hour group interview or 30 minutes one-on-one interview, but participation is voluntary.

6. Do I have to be in the study? Can I withdraw from the study once I've started?

Participation in this study is entirely voluntary. You are not obliged to participate. If you do participate, you can withdraw at any time without having to give any reason and without any penalty. Whatever your decision, it will not affect your relationship with the Hospital, Local Health District and The University of Sydney, or the standard of care you receive now or in the future.

7. Are there any risks or costs associated with being in the study?

Aside from giving up your time to complete three 5-10 minutes surveys (plus a possible 30-60 minutes for an interview if you're interested), we do not expect that there will be any risks or costs associated with taking part in this study.

8. Are there any benefits associated with being in the study?

If you are allocated to receive the new care pathway, you may benefit from having faster access to Physiotherapy and Rheumatology care. You may also improve faster because you are seen sooner. If you are allocated to receive the current care pathway, you receive the same treatment as if you had not taken part in the study.

By participating you will be contributing to important research that helps us understand whether our new pathway is potentially beneficial for people with low back pain and worth investigating in a large future study. The results will help us develop better ways to improve the quality of care provided to patients.

9. What will happen to information about me that is collected during the study?

All data collected will be entered electronically and stored on a research database named REDCap (Research Electronic Data Capture). This is a secure, web-based, non-commercial, data management tool designed for research purposes, hosted and backed up on the Sydney Local Health District servers on a daily basis. No personnel other than the researchers will have access to the research documents. The data will be analysed by the researchers at the Royal Prince Alfred Hospital. All data for use in journal publications and presentations will be de-identified. The files will be retained for 15 years from the day the study is completed. Once this retention expires, the files will be disposed of using the Royal Prince Alfred Hospital confidential waste disposal service.

The data may be used for future research purposes; however, Human Research Ethics Committee (HREC) approval will be sought prior to any future use of the data. It will not be shared with local or international collaborators.

If you are allocated to the new pathway, you may be provided with an exercise program delivered via a mobile App (PhysiTrack). No data will be collected through the PhysiTrack App and therefore no data will be sent to the developer. The App will simply be used to show you which exercises to do. PhysiTrack

is also not a medical device hence does not require TGA approval. PhysiTrack is simply an App that allows physiotherapists to put together an exercise program to allow you to receive written and video instructions on how to perform the exercises correctly. PhysioTrack is essentially a substitute for drawing an exercise program on a piece of paper. The exercises in PhysiTrack include a range of exercises physiotherapists have been prescribing for patients over many years.

As with any home-exercise program prescribed by a physiotherapist, you are free to stop exercising or using the PhysiTrack app at any time if you experience an increase in your symptoms or are not comfortable performing an exercise.

10. Will I be told the results of the study?

You have a right to receive feedback about the overall results of this study. You can tell us that you wish to receive feedback by ticking a box and leaving your email when you complete the consent form. This feedback will be in the form of a one-page lay summary of the results. You will receive this feedback after the study is finished.

11. What do I do next?

When you have read this information, please store it in a safe place. If you understand what you have read and would like to participant, please sign and return the consent form.

If you would like to know more about the study at any stage and ask questions, please feel free to contact Mr Christopher Han (research assistant) at Christopher.Han@sydney.edu.au or (02) 8627 7423.

12. What if I have a complaint or any concerns about the study?

This study has been approved by the Ethics Review Committee (RPAH Zone) of the Sydney Local Health District.

If you have any complaints or concerns about any aspect of this study, you should call our research team who will do their best to address any issues. If your concerns are not able to be addressed, you can contact the Executive Officer of the Ethics Review Committee on 02 9515 7176 and quote protocol number X21-0221.

13. Complaints and compensation

If you suffer any injuries or complications as a result of the research project, you will be advised to contact the study team and will be assisted with arranging appropriate medical treatment. If you are eligible for Medicare, you can receive any medical treatment required to treat the injury or complication, free of charge, as a public patient in any Australian public hospital.

In addition, you may have a right to take legal action to obtain compensation for any injuries or complications resulting from the study. Compensation may be available if your injury or complication is sufficiently serious and is caused by unsafe drugs or equipment, or by the negligence of one of the parties involved in the study (for example, the researcher, the hospital, or the treating doctor). You do not give up any legal rights to compensation by participating in this study.

This information sheet is for you to keep.
