# Supplementary material for: The feasibility of delivering and evaluating stratified care integrated with telehealth (‘Rapid Stratified Telehealth’) for patients with low back pain: a feasibility and pilot randomised controlled trial
Source: Clin Rheumatol. 2026 Apr 7;45(6):3771–84. doi: 10.1007/s10067-026-07955-w (PMC13249632; doi:10.1007/s10067-026-07955-w)
Supplement: Supplementary file 5 — (DOCX 68.7 KB) [file 10067_2026_7955_MOESM5_ESM.docx]

Supplementary file 5: Participant Information Statements for qualitative interviews

| 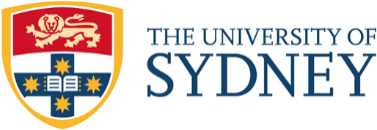 | |  | **School of Public Health Faculty of Medicine and Health** | |
| --- | --- | --- | --- | --- |
|  | ABN 15 211 513 464 | | |  |
|  | **Dr Joshua Zadro**  *Chief Investigator  Research Fellow* | | | Room 10/071  Level 10 North, King George V Building Royal Prince Alfred Hospital  The University of Sydney  NSW 2050 AUSTRALIA  Telephone: +61 2 8627 6782  Facsimile: +61 2 8627 6262  Email: [joshua.zadro@sydney.edu.au](mailto:joshua.zadro@sydney.edu.au)  Web: <http://www.sydney.edu.au> |

**Rapid Virtual Stratified Care for people with back pain and other musculoskeletal conditions: an interview study**

PARTICIPANT INFORMATION STATEMENT - patients

1. **What is this study about?**

You are invited to take part in a research study that will explore people’s opinion on using a screening tool to categorise patients care needs (stratified care) to choose if they are appropriate for telehealth (e.g. Rapid virtual stratified care). This Participant Information Statement tells you about the study. Knowing what is involved will help you decide if you want to take part. Please read this sheet carefully and ask questions about anything that you don’t understand or want to know more about.

Participation in this research study is voluntary.

By giving your consent to take part in this study you are telling us that you:

- Understand what you have read
- Agree to take part in the research study as outlined below
- Agree to the use of your personal information as described

This Participant Information Statement is yours to keep.

Your decision whether to participate will not affect your current or future relationship with the researchers or anyone else at the University of Sydney or Royal Prince Alfred Hospital. It also won’t affect the quality of care you receive.

1. **Who is running the study?**

This study is funded by the Agency for Clinical Innovation (ACI) New South Wales and the National Health Medical Research Council. Neither funder will benefit commercially from this study.

The people conducting this study are:

- Dr Joshua Zadro, NHMRC Postdoctoral Researcher, Institute for Musculoskeletal Health University of Sydney and Sydney Local Health District
- Dr Chris Needs, Staff Specialist Rheumatologist, Royal Prince Alfred Hospital, Sydney Local District Health
- Prof Christopher Maher, Director, Institute for Musculoskeletal Health, University of Sydney and Sydney Local Health District
- Dr David Martens, Rheumatologist Advanced Trainee, Royal Prince Alfred Hospital, Sydney Local District Health
- Ms Danielle Coombs, Physiotherapist, Institute for Musculoskeletal Health University of Sydney and Sydney Local Health District
- Dr Gustavo Machado, NHMRC Postdoctoral Researcher, Institute for Musculoskeletal Health University of Sydney and Sydney Local Health District
- Mrs Charlotte McLennan, Network Manager, Institute for Musculoskeletal Health University of Sydney and Sydney Local Health District
- Dr Cameron Adams, Rheumatologist Advanced Trainee, Royal Prince Alfred Hospital, Sydney Local District Health
- Prof Nadine Foster, Director, Surgical, Treatment and Rehabilitation Service (STARS) Research and Education Alliance, The University of Queensland and Metro North Hospital and Health Service
- Mr Andrew Gamble, Physiotherapist, Institute for Musculoskeletal Health, Sydney School of Public Health, University of Sydney & Sydney Local Health District

1. **Who can take part in the study?**

A person will be allowed to participate in this study if he or she participated in our study comparing two care pathways for people with back pain and/or leg pain radiating from the back and completed the 6 month follow up. People who require treatment for any musculoskeletal condition and are interested in our new model of care are also eligible.

1. **What does the study involve?**

If you agree to participate in our study, we will arrange a time for you to participate in a group interview (with up to 8 other participants who took part in the study) or a one-on-one interview if you prefer. This interview may be conducted via telephone or videoconference (e.g. Zoom) or in person at the Institute for Musculoskeletal Health, Level 10 King George V Building, Royal Prince Alfred Hospital. The interview will explore your opinion on combining the use of stratified care and telehealth (e.g. Rapid virtual stratified care) to manage back pain or other musculoskeletal conditions. The interviews will be audio-recorded and transcribed verbatim for analysis. The recordings will be deleted after they have been transcribed.

1. **How much of my time will the study take?**

If you decide to participate, you will need to participate in a 1 hour group interview or 30 minute one-on-one interview. If you would like the interview to be face-to-face, there may be travel time to get to the Institute for Musculoskeletal Health.

1. **Do I have to be in the study? Can I withdraw from the study once I've started?**

Participation in this study is entirely voluntary. You are not obliged to participate. If you do participate, you can withdraw at any time without having to give any reason and without any penalty. Whatever your decision, it will not affect your relationship with the Hospital, Local Health District and The University of Sydney, or the standard of care you receive now or in the future.

1. **Are there any risks or costs associated with being in the study?**

Aside from giving up your time to participate in an interview, we do not expect that there will be any risks or costs associated with taking part in this study.

1. **Are there any benefits associated with being in the study?**

By participating, you will be contributing to important research that helps us understand whether the new care pathway that we are testing is acceptable to patients with back pain and other musculoskeletal conditions. The results may help us refine the care pathway before testing it in large research study.

1. **What will happen to information about me that is collected during the study?**

By providing your consent, you are agreeing to us collecting personal information about you for the purposes of this research study. Your information will only be used for the purposes outlined in this Participant Information Statement, unless you consent otherwise.

Your information will be stored and analysed securely on a research database within the Institute for Musculoskeletal Health, Sydney Local Health District, and your identity/information will be kept strictly confidential, except as required by law. Study findings may be published, but you will not be individually identifiable in these publications*.*

We will keep the information we collect for this study, and we may use it in future project. By providing your consent you are allowing us to use your information in future projects, however all identifying data will remain strictly confidential. We don’t know at this stage what these other projects may involve. We will seek ethical approval before using the information in these future projects.

1. **Will I be told the results of the study?**

You have a right to receive feedback about the overall results of this study. You can tell us that you wish to receive feedback by ticking a box and leaving your email when you complete the questionnaires. This feedback will be in the form of a one-page lay summary of the results. You will receive this feedback after the study is finished.

1. **What do I do next?**

When you have read this information, please store it in a safe place. If you understand what you have read and would like to participant, please complete the online consent form, or sign and return the paper consent form.

If you would like to know more about the study at any stage and ask questions, please feel free to contact Mr Andrew Gamble (PhD student and research assistant) at [agam1165@uni.sydney.edu.au](mailto:agam1165@uni.sydney.edu.au) or (02) 8627 7423.

1. **What if I have a complaint or any concerns about the study?**

This study has been approved by the Ethics Review Committee (RPAH Zone) of the Sydney Local Health District.

If you have any complaints or concerns about any aspect of this study, you should call our research team who will do their best to address any issues. If your concerns are not able to be addressed, you can contact the Executive Officer of the Ethics Review Committee on 02 9515 6766 and quote protocol number **X21-0221**.

This information sheet is for you to keep.

| 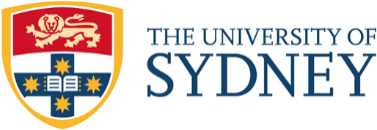 | |  | **School of Public Health Faculty of Medicine and Health** | |
| --- | --- | --- | --- | --- |
|  | ABN 15 211 513 464 | | |  |
|  | **Dr Joshua Zadro**  *Chief Investigator  Research Fellow* | | | Room 10/071  Level 10 North, King George V Building Royal Prince Alfred Hospital  The University of Sydney  NSW 2050 AUSTRALIA  Telephone: +61 2 8627 6782  Facsimile: +61 2 8627 6262  Email: [joshua.zadro@sydney.edu.au](mailto:joshua.zadro@sydney.edu.au)  Web: <http://www.sydney.edu.au> |

**Rapid Virtual Stratified Care for people with back pain and other musculoskeletal conditions: an interview study**

PARTICIPANT INFORMATION STATEMENT – clinicians and key stakeholders

1. **What is this study about?**

You are invited to take part in a research study that will explore clinicians’ opinion on combining stratified care and telehealth (e.g. Rapid virtual stratified care). This Participant Information Statement tells you about the study. Knowing what is involved will help you decide if you want to take part. Please read this sheet carefully and ask questions about anything that you don’t understand or want to know more about.

Participation in this research study is voluntary.

By giving your consent to take part in this study you are telling us that you:

- Understand what you have read
- Agree to take part in the research study as outlined below
- Agree to the use of your personal information as described

This Participant Information Statement is yours to keep.

Your decision whether to participate will not affect your current or future relationship with the researchers or anyone else at the University of Sydney or Royal Prince Alfred Hospital.

1. **Who is running the study?**

This study is funded by the Agency for Clinical Innovation (ACI) New South Wales and the National Health Medical Research Council. Neither funder will benefit commercially from this study.

The people conducting this study are:

- Dr Joshua Zadro, NHMRC Postdoctoral Researcher, Institute for Musculoskeletal Health University of Sydney and Sydney Local Health District
- Dr Chris Needs, Staff Specialist Rheumatologist, Royal Prince Alfred Hospital, Sydney Local District Health
- Prof Christopher Maher, Director, Institute for Musculoskeletal Health, University of Sydney and Sydney Local Health District
- Dr David Martens, Rheumatologist Advanced Trainee, Royal Prince Alfred Hospital, Sydney Local District Health
- Ms Danielle Coombs, Physiotherapist, Institute for Musculoskeletal Health University of Sydney and Sydney Local Health District
- Dr Gustavo Machado, NHMRC Postdoctoral Researcher, Institute for Musculoskeletal Health University of Sydney and Sydney Local Health District
- Mrs Charlotte McLennan, Network Manager, Institute for Musculoskeletal Health University of Sydney and Sydney Local Health District
- Dr Cameron Adams, Rheumatologist Advanced Trainee, Royal Prince Alfred Hospital, Sydney Local District Health
- Prof Nadine Foster, Director, Surgical, Treatment and Rehabilitation Service (STARS) Research and Education Alliance, The University of Queensland and Metro North Hospital and Health Service
- Mr Andrew Gamble, Physiotherapist, Institute for Musculoskeletal Health, Sydney School of Public Health, University of Sydney & Sydney Local Health District

This study is funded by the Agency for Clinical Innovation (ACI) New South Wales and the National Health Medical Research Council. Neither funder will benefit commercially from this study.

1. **Who can take part in the study?**

A person will be allowed to participate in this study if he or she is a physiotherapist or rheumatologist who provided care as part of our study comparing two care pathways for people with back pain and/or leg pain radiating from the back. Physiotherapists or rheumatologists who were not involved in the study and key stakeholders that may be interested in the new model of care are also eligible.

1. **What does the study involve?**

If you agree to participate in our study, we will arrange a time for you to participate in a one-on-one interview with a member of the research team. This interview may be conducted via telephone or videoconference (e.g. Zoom) or in person at the Institute for Musculoskeletal Health, Level 10 King George V Building, Royal Prince Alfred Hospital. The interview will explore your opinion on barriers, facilitators and acceptability of combining stratified care and telehealth (e.g. Rapid virtual stratified care). The interviews will be audio-recorded and transcribed verbatim for analysis. The recordings will be deleted after they have been transcribed.

1. **How much of my time will the study take?**

If you decide to participate, you will need to participate in a 30 minute one-on-one interview. If you would like the interview to be face-to-face, there may be travel time to get to the Institute for Musculoskeletal Health.

1. **Do I have to be in the study? Can I withdraw from the study once I've started?**

Participation in this study is entirely voluntary. You are not obliged to participate. If you do participate, you can withdraw at any time without having to give any reason and without any penalty. Whatever your decision, it will not affect your relationship with the Hospital, Local Health District and The University of Sydney.

1. **Are there any risks or costs associated with being in the study?**

Aside from giving up your time to participate in an interview, we do not expect that there will be any risks or costs associated with taking part in this study.

1. **Are there any benefits associated with being in the study?**

By participating, you will be contributing to important research that helps us understand whether the new care pathway that we are testing is acceptable for patients with back pain and/or leg pain radiating from the back, other musculoskeletal conditions, and clinicians providing care to these patients. The results may help us refine the care pathway before testing it in large research study.

1. **What will happen to information about me that is collected during the study?**

By providing your consent, you are agreeing to us collecting personal information about you for the purposes of this research study. Your information will only be used for the purposes outlined in this Participant Information Statement, unless you consent otherwise.

Your information will be stored and analysed securely on a research database within the Institute for Musculoskeletal Health, Sydney Local Health District, and your identity/information will be kept strictly confidential, except as required by law. Study findings may be published, but you will not be individually identifiable in these publications*.*

We will keep the information we collect for this study, and we may use it in future projects. By providing your consent you are allowing us to use your information in future projects, however all identifying data will remain strictly confidential. We don’t know at this stage what these other projects may involve. We will seek ethical approval before using the information in these future projects.

1. **Will I be told the results of the study?**

You have a right to receive feedback about the overall results of this study. You can tell us that you wish to receive feedback by ticking a box and leaving your email when you complete the questionnaires. This feedback will be in the form of a one-page lay summary of the results. You will receive this feedback after the study is finished.

1. **What do I do next?**

When you have read this information, please store it in a safe place. If you understand what you have read and would like to participant, please complete the online consent form, or sign and return the paper consent form.

If you would like to know more about the study at any stage and ask questions, please feel free to contact Mr Andrew Gamble (PhD student and research assistant) at [agam1165@uni.sydney.edu.au](mailto:agam1165@uni.sydney.edu.au) or (02) 8627 7423.

1. **What if I have a complaint or any concerns about the study?**

This study has been approved by the Ethics Review Committee (RPAH Zone) of the Sydney Local Health District.

If you have any complaints or concerns about any aspect of this study, you should call our research team who will do their best to address any issues. If your concerns are not able to be addressed, you can contact the Executive Officer of the Ethics Review Committee on 02 9515 7176 and quote protocol number **X21-0221**.

This information sheet is for you to keep.
