# Supplementary material for: The feasibility of delivering and evaluating stratified care integrated with telehealth (‘Rapid Stratified Telehealth’) for patients with low back pain: a feasibility and pilot randomised controlled trial
Source: Clin Rheumatol. 2026 Apr 7;45(6):3771–84. doi: 10.1007/s10067-026-07955-w (PMC13249632; doi:10.1007/s10067-026-07955-w)
Supplement: Supplementary file 6 — (DOCX 62.1 KB) [file 10067_2026_7955_MOESM6_ESM.docx]

Supplementary file 6: Participant consent forms for qualitative interviews

**Rapid Virtual Stratified Care for people with back pain and other musculoskeletal conditions: an interview study**PARTICIPANT CONSENT FORM - patients

I, ................................................................................................................ [print name], agree to take part in the research project:

In giving my consent I acknowledge that:

| Please tick/initial boxes | |
| --- | --- |
| 🞏 | I have read the Participant Information Statement and have been given the opportunity to discuss the study and my involvement in it with the researcher/s. |
| 🞏 | The procedures required and time involved (including any inconvenience, risk, discomfort or side effect, and their implications) have been explained to me, and my questions about the project have been answered to my satisfaction. |
| 🞏 | I understand that participation is voluntary. I am under no obligation to consent. |
| 🞏 | I understand that I can withdraw from the study at any time, without providing a reason and without suffering any penalty. This will not affect my relationship with the researcher/s, university, Sydney Local District Health, or RPA Hospital. |
| 🞏 | The interview I participate in will be audio-recorded and transcribed verbatim. I understand that I can stop my participation at any time and select to erase the interview to that point. |
| 🞏 | I understand that my involvement is strictly confidential and no information about me will be used in any way that reveals my identity. |
| 🞏 | I understand that data from this study may be used again for future research purposes, but that all data is strictly confidential and no information about me will be used in any way that reveals my identity. |
| 🞏 | I would like the researchers to contact me to inform me about the results of the study. |

Signature:_________________________________________________________________________

Date:_____________________________________________________________________________

You have a right to receive feedback about the overall results of this study. This feedback will be a one-page summary regarding the overall findings of the study.

**Would you like to receive feedback about the overall results of this study?**

- Yes
- No

If yes, please provide your email address ________________________

This study has been approved by the Ethics Review Committee (RPAH Zone) of the Sydney Local Health District. Any person with concerns or complaints about the conduct of this study should contact the Executive Officer on 02 9515 6766 and quote protocol number X21-0221.

| 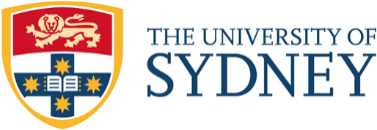 | |  | **School of Public Health Faculty of Medicine and Health** | |
| --- | --- | --- | --- | --- |
|  | ABN 15 211 513 464 | | |  |
|  | **Dr Joshua Zadro**  *Chief Investigator  Research Fellow* | | | Room 10/071  Level 10 North, King George V Building Royal Prince Alfred Hospital  The University of Sydney  NSW 2050 AUSTRALIA  Telephone: +61 2 8627 6782  Facsimile: +61 2 8627 6262  Email: [joshua.zadro@sydney.edu.au](mailto:joshua.zadro@sydney.edu.au)  Web: <http://www.sydney.edu.au> |

**Rapid Virtual Stratified Care for people with back pain and other musculoskeletal conditions: an interview study**

**PARTICIPANT CONSENT FORM - clinicians and key stakeholders**

I,_____________________________________________________________________ *[full name]*

Of_____________________________________________________________________ *[address]*

have read and understood the Participant Information Sheet on the abovenamed research study

and have discussed the study with ___________________________________________________ [investigator responsible for conducting informed consent].

- I have been made aware of the procedures involved in the study, including any known or expected inconvenience, risk, discomfort or potential side effect and of their implications as far as they are currently known by the researchers.
- I understand that the interview discussion will be audio-recorded and will then be transcribed and be kept in a manner in which I cannot be identified for analysis and I agree to this.
- I understand that my de-identified data may be used for future research and I agree to this.
- I would like to receive a copy of the study results when they become available. My email address

  is: __________________________________________________________________________
- I understand that, during the course of this study, my medical records may be accessed by Sydney Local Health District by regulatory authorities or by the Ethics Committee approving the research in order to verify results and determine that the study is being carried out correctly.
- I understand that the SLHD software license for REDCap (Research Electronic Data Capture) will be used to manage the collection and storage of my research data.
- I have had an opportunity to ask questions and I am satisfied with the answers I have received.
- I freely choose to participate in this study and understand that I can withdraw at any time.
- I consent to the future use of any data / samples I provide for research purposes. I understand that before they can use any data I provide, they must seek additional ethics approval. YES/ NO
- I consent for other research collaborators to use any data / samples I provide for future research purposes. I understand that before they can use my data, they must seek additional ethics approval. YES/NO
- I also understand that the research study is strictly confidential.
- I hereby agree to participate in this research study.
- I consent to the storage and use of my information collected from me for use, as described in the relevant section of the Participant Information Sheet, for:

-This specific research project

-Other research that is closely related to this research project

-Any future research

Participant Name:_____________________________________________­­____________­________

Participant Signature:______________________________________________________________

Date:__________________________________________________________________________

Name of Person conducting informed consent:__________________________________________

Signature of Person conducting informed consent: ______________________________________

Date:___________________________________________________________________________
