# Supplementary material for: The feasibility of delivering and evaluating stratified care integrated with telehealth (‘Rapid Stratified Telehealth’) for patients with low back pain: a feasibility and pilot randomised controlled trial
Source: Clin Rheumatol. 2026 Apr 7;45(6):3771–84. doi: 10.1007/s10067-026-07955-w (PMC13249632; doi:10.1007/s10067-026-07955-w)
Supplement: Supplementary file 7 — (DOCX 38.0 KB) [file 10067_2026_7955_MOESM7_ESM.docx]

Supplementary file 7: Semi-structured interview guides

**INTERVIEW GUIDE FOR PATIENTS INCLUDED IN TRIAL**

***Questions do not have to be asked in this order, and not all questions have to be covered***

**Introduction**

Hi, my name is [name]. Thank you for taking part in this interview. Researchers and health professionals at The University of Sydney and Royal Prince Alfred Hospital want to find out whether a new treatment pathway using telephone and virtual appointments, and App-based exercise programs, helps people receive treatment sooner and get better sooner.

We would like to ask you questions about the treatment you received in the Back Clinic. If at any time you would like to stop the interview, please let us know and we will stop. You can change your mind about talking to me at any time before or during the interview and stop the interview at any time. You can choose not to answer a question.

Are you happy to continue? [If no, thank them for their time and end the interview; if yes, continue].

Thank you [name] for agreeing to take part. We will use your feedback and the feedback of others to write a summary of what people have told us. There will be absolutely no identification of any real names or identification of where you live or which hospitals or health professionals you have seen.

Are you happy for me to record the interview? Do you have any questions before we start?

**CONTEXT:** TO UNDERSTAND WHAT WORKED, WHAT DIDN’T WORK, AND WHY/ WHY NOT FOR THE TWO METHODS OF SERVICE DELIVERY.

I am interested in exploring your experiences with the care you received in greater detail. Please feel free to be honest about what it was like for you.

***All participants***

1. Please tell me about your experiences overall of [face-to-face care, virtual consultation, App, pain education program, telephone consultation].

Prompts:

- What aspects of the experience do you like most, and why?
- What do you like least, and why?

2. Was your treatment convenient?

Prompts:

- How convenient was it for you to receive [face-to-face care, virtual consultation, App, pain education program, telephone consultation]?
- How do you feel about not having to attend the hospital for treatment (for low-, medium-, and high-risk participants)?
- How do you feel about having to attend the hospital for treatment (for participants with worrisome radicular symptoms and the usual care group)?

***Low-risk participants***

3. Next, I’d like to get your views about the virtual/telephone call you received (or why you did not receive it).

Prompts:

- How did you find the call? What was helpful? What wasn’t?
- Do you feel as though you got any benefit from the phone call?
- What kinds of things did you talk about with the rheumatologist?
- Would you recommend this method of delivering for others? What kinds of people would this approach suit? Who wouldn’t it suit?
- What else would you liked to have received as part of your treatment during the trial?

***Medium- and high risk participants***

4. Next, I’d like to get your views about the virtual consultation(s) you received (or why you did not receive them).

Prompts:

- How did you find the consultation(s)? What was helpful? What wasn’t?
- Do you feel as though you got any benefit from the virtual consultation(s)?
- Do you feel the benefit was similar to what you would have got with face-to-face appointment(s)?
- Would you recommend this method of delivering treatment for others? What kinds of people would this approach suit? Who wouldn’t it suit?
- What else would you liked to have received as part of your treatment during the trial?
- Can you comment on the frequency of your appointments?

5. Next, I want to discuss the PhysiTrack App.

- Did you ever use the App?
- If no, why was that?
- If yes, how easy was it to use the App? Did it get easier over time?
- Did you need help to use it? If yes, explore.
- What do you think about the physio using the App to monitor your compliance with the rehabilitation exercises? Why do you say that?
- Did your recording of exercises match what exercise you did?
- How long did you use the App?
- How long did you do the rehabilitation exercises? Why or why not?

6. Next, I want to discuss the self-directed pain education program. **(High risk participants)**

- Did you access the program?
- If no, why was that?
- If yes, how easy was it to navigate? Did it get easier over time?
- Did you need help to access it? If yes, explore.
- How did you find the information in the program?
- Did you watch all the videos? Explore

***Participants with worrisome radicular pain (and people in the usual-care group)***

7. Next, I’d like to get your views about the face-to-face appointments you received (or why you did not receive them).

Prompts:

- How did you find the appointment(s)? What was helpful? What wasn’t?
- Can you tell me about the process of scheduling appointments? What was the availability of your rheumatologist and physiotherapist?
- Did you always have the same person?
- What is it about seeing a rheumatologist or physiotherapist in person that you like or don’t like?
- How convenient was it for you to travel to and attend a face-to-face appointment(s) at the hospital?
- Can you comment on the frequency of your appointments? Is that what you expected? Why or why not?
- Do you feel as though you got any benefit from the appointment(s)?
- Would you recommend this method of delivering treatment for others? What kinds of people would this approach suit? Who wouldn’t it suit?
- Do you feel you could have a got a similar benefit from a telephone or virtual consultation(s)?
- What else would you liked to have received as part of your treatment during the trial?

8. Is there anything else you would like to say that we have not talked about in this interview?

Thank you so much for your time.

**INTERVIEW GUIDE FOR EXTERNAL PATIENTS**

***Questions do not have to be asked in this order, and not all questions have to be covered***

**Introduction**

Hi, my name is [name]. Thank you for taking part in this interview. Researchers and health professionals at The University of Sydney and Royal Prince Alfred Hospital want to find out whether a new treatment pathway using telephone and virtual appointments, and App-based exercise programs, helps people receive treatment sooner and get better sooner.

We would like to ask you questions about your thoughts on the model of care (rapid virtual care) and our trial testing this model. If at any time you would like to stop the interview, please let us know and we will stop. You can change your mind about talking to me at any time before or during the interview and stop the interview at any time. You can choose not to answer a question.

Are you happy to continue? [If no, thank them for their time and end the interview; if yes, continue].

Thank you [name] for agreeing to take part. We will use your feedback and the feedback of others to write a summary of what people have told us. There will be absolutely no identification of any real names or identification of where you live or which hospitals or health professionals you have seen.

Are you happy for me to record the interview? Do you have any questions before we start?

***All participants***

*Participants will be asked about basic demographic information and other questions to give further background on relevant characteristics (e.g., what is their current musculoskeletal condition).*

*1. What are your thoughts on the positives and negatives of this model of care?*

*2. What reason or reasons would (did) you decide to not participate in the trial testing this model?*

*3. What reason or reasons would (did) you not provide follow-up data (or stop providing data)?*

*Prompts*

- *Explanation of the model of care*
- *Ask participants on their thoughts on what could be improved to make the model more suitable for them and more likely to participate*
- *Ask participants what information they would need to hear during the recruitment phone call to increase the likelihood they would participate and complete follow up data*

*4. Do you have any other musculoskeletal pain?*

- *If yes, do you think this model of care will be beneficial for this pain if you would otherwise see a physiotherapist or rheumatologist?*

5. Is there anything else you would like to say that we have not talked about in this interview?

Thank you so much for your time.

**INTERVIEW GUIDE FOR CLINICIANS INCLUDED IN THE TRIAL**

(Questions do not have to be asked in this order, and not all questions have to be covered.)

Hi, my name is [name and background]. Thank you for taking part in this interview. Researchers and health professionals at The University of Sydney and Royal Prince Alfred Hospital want to find out whether a new treatment pathway using telephone and virtual appointments, and App-based exercise programs, helps people receive treatment sooner and get better sooner. We also want to see if the new treatment pathway is acceptable to clinicians.

We would like to ask you questions about the treatment you provided in the Back Clinic as part of the trial. You can change your mind about talking to me at any time before or during the interview and stop the interview at any time.

Are you happy to continue? [If no, thank them for their time and end interview; if yes continue.]

Thank you [name] for agreeing to take part. We will use your feedback and the feedback of others to write a summary of what people have told us. There will be absolutely no identification of any real names or identification of your professional details.

Are you happy for me to record the interview? Do you have any questions before we start?

CONTEXT: TO UNDERSTAND WHAT WORKED, WHAT DIDN’T WORK, AND WHY/ WHY NOT FOR THE TWO METHODS OF SERVICE DELIVERY

*Participants will be asked about basic demographic information and other questions to give further background on relevant characteristics (e.g., hospital site, professional experience, years in current position and years since graduated).*

1. Let’s first talk about the way your service normally operates.

Prompt:

- How often would you typically see patients? Do you have a waiting list? How long is that waiting list usually?

2. Please tell me about your overall experiences coordinating the Rapid Virtual Stratified Care trial.

Prompts:

- What pleased you about the trial?
- What surprised you?
- What were your concerns?
- What would you do differently?

3. How did the clinicians and patients involved in the trial respond to being involved?

4. Please tell us about the recruitment process for the trial.

Prompts:

- How did you manage the logistics of recruitment?
- Was there any difficulty in recruiting participants? If so, please describe.

CONTEXT: TO UNDERSTAND THE PERCEIVED BARRIERS/ FACILITATORS FOR EVALUATING IN A LARGE, MULTI-SITE TRIAL

5. On the basis of your experience in the trial, how easy do you think it will be to introduce delivering this model of care in other outpatient musculoskeletal settings?

6. Has the COVID-19 crisis changed your or your colleagues’ attitudes towards delivering rehabilitation remotely?

7. Looking back on the approach used to deliver treatment using eHealth in the trial – are there any aspects of the intervention that could have been delivered differently?

Prompts:

- Could participants at high-risk of persistent pain be better managed with face-to-face appointments?
- Could participants with worrisome radicular symptoms be managed equally effectively with virtual appointments?

8. What is the potential for eHealth-based stratified care to provide more patients with treatment sooner? How important is it to cut down waiting lists?

9. Thinking about what you have learnt from your experiences in the trial – what are the pros and cons of using eHealth-based stratified care, from patients’ perspectives?

Prompts:

- What are the main advantages for patients compared to usual practice?
- How acceptable is eHealth-based stratified care likely to be to those accessing treatment for low back pain in a public hospital? Why or why not?
- What kinds of patients do you think are most suitable for being managed or monitored using eHealth?

10. What are the pros and cons from a clinician’s perspective?

Prompts:

- How compatible/ acceptable will eHealth-based stratified care be to hospital physios and rheumatologists?
- What are the main advantages for clinicians in delivering care via eHealth, compared to usual practice? What are the main disadvantages?

11. What has to be in place for eHealth-based stratified care to be viable to deliver in the hospital setting?

Prompts:

- What are some things that will make this hard/ easy?
- Could this model of care be rolled out in your hospital right now?
- What are some of the barriers?
- What are some of the facilitators?
- Where will the main resistance come from?

12. What kinds of benefits would you anticipate that introducing eHealth-based stratified care would have for patients; physiotherapists; rheumatologists; for hospitals? (Ask about health, service access, cost savings for the hospital).

13. If eHealth-based stratified care was found to be beneficial in a large trial, would you want to provide this intervention in the future? Why, or why not?

14. Is there anything else you would like to say that we have not talked about in this interview?

Thank you so much for your time.

**INTERVIEW GUIDE FOR EXTERNAL CLINICIANS AND KEY STAKEHOLDERS**

(Questions do not have to be asked in this order, and not all questions have to be covered.)

Hi, my name is [name and background]. Thank you for taking part in this interview. Researchers and health professionals at The University of Sydney and Royal Prince Alfred Hospital want to find out whether a new treatment pathway using telephone and virtual appointments, and App-based exercise programs, helps people receive treatment sooner and get better sooner. We also want to see if the new treatment pathway is acceptable to clinicians.

We would like to ask you questions about the model of care we used in the Back Clinic as part of a trial and the potential of combining stratified care and telehealth to treat various musculoskeletal condition. You can change your mind about talking to me at any time before or during the interview and stop the interview at any time.

Are you happy to continue? [If no, thank them for their time and end interview; if yes continue.]

Thank you [name] for agreeing to take part. We will use your feedback and the feedback of others to write a summary of what people have told us. There will be absolutely no identification of any real names or identification of your professional details.

Are you happy for me to record the interview? Do you have any questions before we start?

CONTEXT: TO UNDERSTAND THE PERCEIVED BARRIERS/ FACILITATORS FOR EVALUATING IN A LARGE, MULTI-SITE TRIAL

*Participants will be asked about basic demographic information and other questions to give further background on relevant characteristics (e.g., hospital site, professional experience, years in current position and years since graduated where relevant)*

1. Let’s first talk about the way your service normally operates.

Prompt:

- How often would you typically see patients? Do you have a waiting list? How long is that waiting list usually?

2. Based on your knowledge, how easy do you think it will be to introduce delivering this model of care in other outpatient musculoskeletal settings?

Prompt:

- *Explanation of the model of care*

3. Has the COVID-19 crisis changed your or your colleagues’ attitudes towards delivering rehabilitation remotely?

4. Looking at the approach used to deliver treatment using eHealth in our trial – are there any aspects of the intervention that could have been delivered differently?

Prompts:

- Could participants at high-risk of persistent pain be better managed with face-to-face appointments?
- Could participants with worrisome radicular symptoms be managed equally effectively with virtual appointments?

5. What is the potential for eHealth-based stratified care to provide more patients with treatment sooner? How important is it to cut down waiting lists?

6. Thinking about our model of care – what do you think the pros and cons of using eHealth-based stratified care would be, from patients’ perspectives?

Prompts:

- What would be the main advantages for patients compared to usual practice?
- How acceptable would eHealth-based stratified care likely be to those accessing treatment for low back pain in a public hospital? Why or why not?
- What kinds of patients do you think are most suitable for being managed or monitored using eHealth?

7. What are the pros and cons from a clinician’s perspective?

Prompts:

- How compatible/ acceptable will eHealth-based stratified care be to hospital physios and rheumatologists?
- What are the main advantages for clinicians in delivering care via eHealth, compared to usual practice? What are the main disadvantages?

8. What has to be in place for eHealth-based stratified care to be viable to deliver in the hospital setting?

Prompts:

- What are some things that will make this hard/ easy?
- Could this model of care be rolled out in your hospital right now?
- What are some of the barriers?
- What are some of the facilitators?
- Where will the main resistance come from?

9. What kinds of benefits would you anticipate that introducing eHealth-based stratified care would have for patients; physiotherapists; rheumatologists; for hospitals? (Ask about health, service access, cost savings for the hospital).

10. If eHealth-based stratified care was found to be beneficial in a large trial, would you want to provide this intervention in the future? Why, or why not?

11. Is there anything else you would like to say that we have not talked about in this interview?

Thank you so much for your time.
