# Supplementary material for: The feasibility of delivering and evaluating stratified care integrated with telehealth (‘Rapid Stratified Telehealth’) for patients with low back pain: a feasibility and pilot randomised controlled trial
Source: Clin Rheumatol. 2026 Apr 7;45(6):3771–84. doi: 10.1007/s10067-026-07955-w (PMC13249632; doi:10.1007/s10067-026-07955-w)
Supplement: Supplementary file 8 — (DOCX 17.4 KB) [file 10067_2026_7955_MOESM8_ESM.docx]

Supplementary file 8: Characteristics of interviewed participants

| **Patients** | **Total sample**  **(n=11)** | **Intervention**  **(n=7)** | **Usual care**  **(n=4)** |
| --- | --- | --- | --- |
| Age, median (IQR)^a^ | 60 (50 to 66) ^b^ | 63 (59 to 66)^d^ | 50 (25 to 70) ^c^ |
| Female, n (%) | 7 (64) | 4 (57) | 3 (75) |
| Language other than English spoken at home, n (%) | 4 (36) | 3 (43) | 1 (25) |
| Employment, n (%) |  |  |  |
| Currently employed | 3 (27) | 3 (43) | 2 (50) |
| Not currently employed | 6 (55) | 4 (57) | 0 (0) |
| Student | 2 (18) | 0 (0) | 2 (5) |
| Unpaid carer | 0 (0) | 0 (0) | 0 (0) |
| Education, n (%) |  |  |  |
| High school (not completed) | 2 (18) | 2 (29) | 0 (0) |
| High school (completed) | 1 (9) | 0 (0) | 1 (25) |
| TAFE/Trade | 3 (27) | 1 (14) | 2 (50) |
| University – postgraduate degree | 1 (9) | 1 (14) | 0 (0) |
| University – undergraduate degree | 4 (36) | 3 (43) | 1 (25) |
| Other | 0 (0) | 0 (0) | 0 (0) |
| Symptom duration of 12 weeks or longer, n (%) | 11 (100) | 7 (100) | 4 (100) |
| Taken sick leave, n (%) | 6 (55) | 5 (71) | 1 (25) |
| Risk subgroup, n (%) |  |  |  |
| Low risk | 1 (9) | 0 (0) | 1 (25) |
| Medium risk | 2 (18) | 1 (14) | 1 (25) |
| High risk | 2 (29) | 2 (29) | 0 (0) |
| Keele STarT MSK score (0-12), median (IQR) | 9 (7 to 10) | 10 (9 to 11) | 5 (3 to 7) |
| Potential radiculopathy | 6 (55) | 4 (57) | 2 (50) |
| **Clinicians** | **Total** (n=2) | | |
| Age, median (IQR) | 50 (40 to 60) | | |
| Female, n (%) | 1 (50%) | | |
| Hospital |  | | |
| Royal Prince Alfred | 2 (100) | | |
| Type of Health profession |  | | |
| Rheumatologist | 1 (50) | | |
| Physiotherapist | 1 (50) | | |
| Years experience, median (IQR) | 20 (12 to 28) | | |
| Years in current position, median (IQR) | 5 (3 to 6) | | |

^a,b,c^ Data missing for two participants age (one from each group). N, Number of participants; IQR, interquartile range; TAFE, Technical and Further Education, MSK, Musculoskeletal.
